# Fractal nematic colloids

**DOI:** 10.1038/ncomms14026

**Published:** 2017-01-24

**Authors:** S. M. Hashemi, U. Jagodič, M. R. Mozaffari, M. R. Ejtehadi, I. Muševič, M. Ravnik

**Affiliations:** 1 Department of Physics, Faculty of Mathematics and Physics, University of Ljubljana, Jadranska 19, 1000 Ljubljana, Slovenia; 2 Department of Physics, Sharif University of Technology, P.O. Box 11155-9161, Tehran 1458889694, Iran; 3 Department of Condensed Matter Physics, Jozef Stefan Institute, Jamova 39, 1000 Ljubljana, Slovenia; 4 Department of Physics, University of Qom, P.O. Box 3716146611, Qom, Iran; 5 Center of Excellence in Complex Systems and Condensed Matter (CSCM), Sharif University of Technology, Tehran 1458889694, Iran

## Abstract

Fractals are remarkable examples of self-similarity where a structure or dynamic pattern is repeated over multiple spatial or time scales. However, little is known about how fractal stimuli such as fractal surfaces interact with their local environment if it exhibits order. Here we show geometry-induced formation of fractal defect states in Koch nematic colloids, exhibiting fractal self-similarity better than 90% over three orders of magnitude in the length scales, from micrometers to nanometres. We produce polymer Koch-shaped hollow colloidal prisms of three successive fractal iterations by direct laser writing, and characterize their coupling with the nematic by polarization microscopy and numerical modelling. Explicit generation of topological defect pairs is found, with the number of defects following exponential-law dependence and reaching few 100 already at fractal iteration four. This work demonstrates a route for generation of fractal topological defect states in responsive soft matter.

## Abstract (web summary)

Topological defects in nematic liquid crystals can be controlled upon external stimuli for designing novel photonic materials. Here, Hashemi *et al*. show geometry-induced formation of fractal defects in nematic fluids with the fractal self-similarity more than 90% from micrometers to nanometres.

Self-similar fractals are characterized by the repeating pattern—for example, of its structure or dynamic behaviour—over a broad range of spatial, time or other scales. The concept of fractality is especially strong in describing complex physical systems that exhibit irregular distributions, for example, of its parts or constituents, and a degree of self-similarity. Some well-known examples of fractals include Brownian motion^1^, polymer networks^2^,^3^, aggregation growth phenomena^4^,^5^, porous media^2^,^6^, glasses^7^, brain networks^8^,^9^, structural details of genomes^10^ and complex dynamics in human physiology^11^. Distinctly geometrical fractals are characterized by the self-similarity of a system to a part of itself across different length scales^6^,^7^, which in case of an ideally self-similar fractal means that the system is invariant over all length scales and has no characteristic length scale. Geometrical fractal patterns of different scaling behaviours are generally determined by the fractal dimension *D*, which quantifies the change in the geometrical details of the fractal relative to the change in the scales^7^. One of the widely studied geometrical fractal shapes that can be introduced by a simple deterministic iterative method is the Koch fractal, often associated with the shape of the ‘Koch snowflake'^12^,^13^, for which all scaling laws and self-similarity features are given by well-defined geometry-based rules. The resolution of Koch fractals is determined by the fractal iteration, which increases by 1 for each fractal refinement of the particle shape. And it is by varying the fractal iteration of the Koch construction that the response of surrounding material at different length scales can be explored.

Fractal properties of liquid crystals were studied in different contexts, including fractal morphology of polymer dispersed liquid crystals^14^, fractal distribution and growth of bend-core, calamitic and doped liquid crystal aggregations^15^,^16^,^17^,^18^, glass phases of liquid crystals inside a porous medium with a random fractal distribution^19^ and fractal-like disordering in smectic liquid crystals^20^,^21^. However, all these studies were primarily focused on understanding and explaining the bulk phase formation, and not the actual microscopic response of the nematic order to a fractal stimulus. Nematic fluids^22^ are characterized by the orientational order of its constituents—typically rod-like or disk-like molecules or particles—that is well responsive to external stimuli, including electric and magnetic fields and surface ordering fields, known as surface anchoring. Presently, the major focus in liquid crystal research is on generation of topological states of the nematic, such as topological handlebody colloids^23^, topological defects as templates for molecular self-assembly^24^, active colloids^25^ and knotted particles^26^, with main motivation to use their inherent birefringence for novel highly-tunable photonic materials and metamaterials^27^,^28^,^29^. In these systems, topological characteristics of objects, and topology in general, is explored as the prime route for designing complexity of the structures, but much less focus is given to the fundamental role of the geometry^30^. Therefore, a question arises as to what extent an irregular and self-similar object, like a fractal shaped particle, can imprint its geometric characteristic features into topological states of the nematic anisotropic environment.

In this article, we demonstrate fractal nematic colloids, revealing the response of anisotropic environment characterized by the nematic ordering field to a fractal surface. Specifically, we explore Koch-fractal-shaped particles of iterations 0 to 3 (and numerically, up to 4) that are shown to induce formation of fractal topological states. Experimentally, direct laser writing into polymer is used for production and polarization microscopy for characterization of these fractal nematic states, whereas theoretically, extensive finite-element modelling is applied, used also as prediction tool for the experiments. The topological states are characterized by exploring the topological defects pairs of opposite charge, with their number increasing exponentially with the fractal iteration. The fractal feature size relative to the nematic correlation length is shown to affect the structure of defects, especially the defect cores, where effective fusing of defect cores is observed at fractal feature sizes comparable to the correlation length, also leading to symmetry breaking of the nematic orientational ordering. Finally, we introduce basic self-similarity functions—local and global—that can be used to characterize fractal self-similarity at different resolution levels in anisotropic nematic environment, finding a window of 2–3 orders of magnitude in length scales where good self-similar response of nematic is observed.

## Results

### Construction of fractal nematic colloids

**Figure 1:**
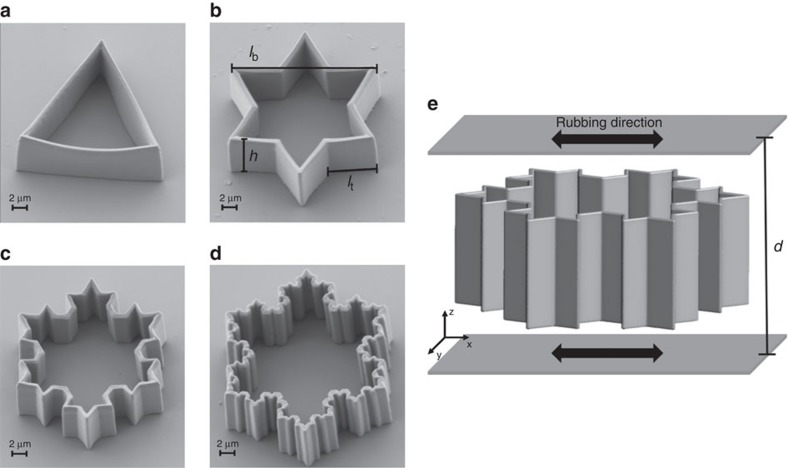
Koch-star fractal particles. (**a**–**d**) Scanning electron microscopy (SEM) images of the zeroth to the third iteration of the Koch star particles that are direct laser-written into a photosensitive polymer by the two-photon laser-induced polymerization (Photonic Professional system by Nanoscribe GmbH). The thickness of the sides of all Koch particles is 0.6 μm, the height is 8 μm and the overall size is 20 μm. The parameters *l*_t_ and *l*_b_ are the side lengths of the smallest and largest equilateral triangles that are used in the geometry construction based on the Koch iterative construction rule. The parameter *h* is the particle height. (**e**) Schematic view of the Koch particle of the second literation in a planar nematic liquid crystal cell. The rubbing directions specify the orientation of LC molecules at the cell surfaces.

To explore the topological properties of a nematic field induced by a fractal geometry, we choose the iteration of Koch fractals. We construct particles with a Koch-fractal-shaped cross section and a thin wall of the height *h*=*l*_b_/2, as shown in the schematic Fig. 1b. The zeroth Koch iteration has a three-fold rotational symmetry axis and the higher Koch iterations have a six-fold rotational symmetry axis.

Real Koch star particles were produced by using the 3D two-photon direct laser writing technique (for more see Supplementary Note 1; Supplementary Fig. 1). Scanning electron microscopy images of the four iterations of the particles are shown in Fig. 1a–d, demonstrating perfect shape and surface smoothness of the polymer particles. The surfaces of the particles were treated with *N*,*N*-dimethyl-*N*-octadecyl-3-aminopropyl trimethoxysilyl chloride (DMOAP) silane (ABCR GmbH) to create perpendicular (homeotropic) surface alignment of liquid crystal molecules (Supplementary Note 1). The particles were dispersed in a low birefringent liquid crystal mixture of CCN-47 (50%) and CCN-55 (50%) (Nematel GmbH), with the nematic to isotropic transition at 65 °C. We enclose the nematic colloidal dispersion in a 30 μm glass cell with strong planar and unidirectional surface alignment. Because of opposing surface alignment on particles and cell's surfaces, the particles are levitated by the force of elastic distortion in the middle of the cell, as illustrated in Fig. 1e.

The Landau-de Gennes numerical modelling of the system is based on the free energy expression for a nematic system by Landau and de Gennes in the fully tensorial form ref. 22 (Supplementary Note 2; Supplementary Fig. 2). To numerically minimize the free energy we use a custom-developed finite-element method, which is capable of scanning the finest structures of a surface with a high resolution. In finite-element method, a surface with its exact mathematical meaning (zero thickness) can be introduced and accordingly the surface-anchoring condition can be unambiguously determined. This makes the finite-element method a powerful technique for the numerical study of systems containing finely-structured and complicated surfaces and is crucially needed in modelling of our fractal colloids. The sharp edges are numerically rounded in order to achieve a more efficient numerical minimization and more realistically reproduce the direct laser written real particles (for more on surface sharpness, see Supplementary Note 2; Supplementary Fig. 3).

Nematic dispersions of Koch star colloidal particles in planar cells were observed with a polarization microscope and the laser tweezers were used to trap, move and manipulate the particles and its topological defects (Supplementary Note 3)^31^,^32^.

### Characterization of nematic field response to fractal surface

**Figure 2:**
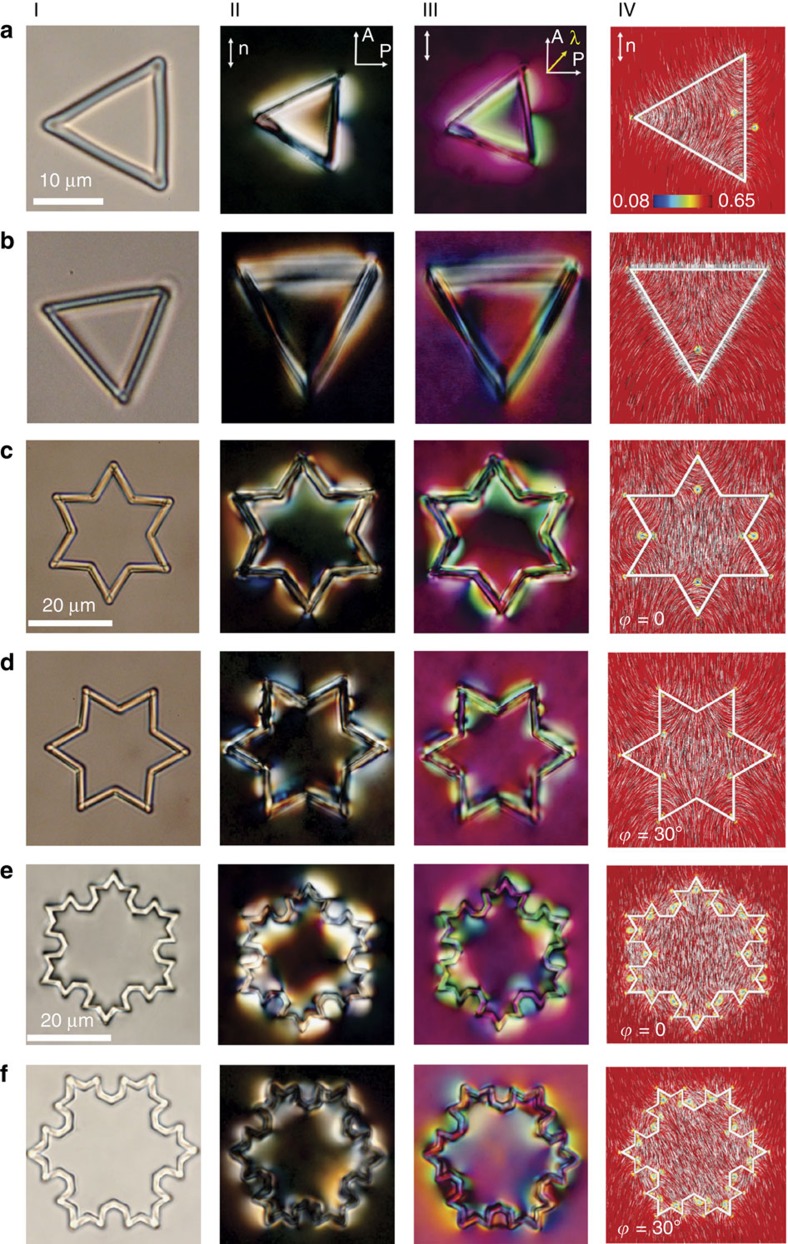
Nematic topological states stabilized by fractal Koch-star colloidal particles. (**a**, I–**f**, I) Koch-star particles in the isotropic phase of the CCN mixture at 70 °C in unpolarized light. (**a**, II–**f**, II) The same particles as in panels I, now observed between crossed polarizers and at room temperature. The rubbing direction, indicating the far-field planar orientation of the nematic is shown by double-headed arrows in (**a**, II–IV). Defects are recognized as point regions in the optical image, surrounded by rapidly varying colour and intensity of the transmitted light, indicating strong director distortion. (**a**, III–**f**, III) The same particles as in **a**, II–**f**, II, now viewed between crossed polarizers and red plate added at 45°. Different colours are due to different in-plane orientations of the nematic molecules. (**a**, IV–**f**, IV) Landau-de Gennes (LdG) numerical modelling illustrating contour plots of the scalar order parameter in the mid-plane of the particles with *l*_b_/*ξ*=100 where *ξ* is the correlation length of the liquid crystal molecules in the *x*–*y* coordinate plane containing the coordinate center. The calculated director field in the *x*–*y* plane of the contour plots is also superposed.

The experimental images of Koch-star nematic colloids are presented in Fig. 2a–f. In the isotropic phase (panels I of Fig. 2a–f) the Koch particles are freely floating in the isotropic melt and could be rotated by liquid flow. Optical artefacts due to diffraction of light from index miss-matching of the Koch star polymer (refractive index 1.5) and the average refractive index of the isotropic phase (1.52) are visible and help to discriminate between the real topological defects in the nematic phase and optical artefacts.

Panels II of Fig. 2a–f show the Koch star particles at room temperature, as observed between crossed polarizers. The zero-iteration Koch particles preferentially orient into two possibilities, that is, with one side parallel or one side perpendicular to the rubbing direction, as shown in panels II to IV in Fig. 2a,b. For the parallel orientation, there are two defects located in the middle of this side, whereas for the perpendicular orientation, there is one defect next to one of the inside corners. Due to elastic repulsion from the confining plates these particles levitate in the middle of the cell and do not tilt or sediment. From the polarized image of this particle in Fig. 2a, II (and similarly also for the particle in Fig. 2b, II) one can clearly see strong director distortions in the corners of the particles. By rotating the analyser at fixed polarizers (Supplementary Fig. 4) one can clearly resolve that defects in the corners are actually pairs of defects with opposite topological winding and charge. Each of the three pairs of defects in each corner of the triangle therefore compensates the winding, giving total winding zero, as expected for the total charge of a torus. One should remember that any iteration of the Koch star particles is topologically equivalent to the torus. Toroidal particles have a genus *g*=1 and it is known from Gauss–Bonnet theorem^24^ that a colloidal handlebody with genus *g* carries defects with a total topological charge of ±(1−*g*). All Koch star particles should therefore have an even number of topological defects which mutually compensate their winding and charge to keep the total charge of any Koch star particle zero at all times.

The first iteration Koch star particles also show two different orientations in the planar cell, rotated at *ϕ*=0 and *ϕ*=30° relative to the rubbing direction as shown in Fig. 2c,d, respectively (and Supplementary Fig. 5). In both cases, polarized and red-plate images show strongly distorted director in the inner and outer corners of the Koch particle, which is the signature of topological defects. By using the laser tweezers it is not possible to detach any defect line (from modelling seen to be running all along the edge of the particle); however, one is able to pull the defects away from the surface. By counting the number of defects, one can see in Fig. 2c, II; c, III eight pairs of defects in the corners, four inner corners do not show any defect. The other configuration of the first iteration Koch particle shown in Fig. 2d, II; d, III, shows six pairs of defects.

The second iteration Koch star particles show again two stable orientations in the planar nematic cell, as shown in Fig. 2e,f and Supplementary Figs 6 and 7. The configuration in Fig. 2e occurs with 70% probability and the symmetry axis of the Koch particle is parallel to the overall orientation of the nematic. Defects of the second iteration Koch particles are different for these two different orientations. They are identified by taking polarized images of the particle at different orientations of the analyser, where the polarizer is kept perpendicular to the overall nematic director, thus exciting the ordinary ray in the cell. Supplementary Figs 6 and 7 show detailed analysis of defects of the second-iteration Koch particles with orientation equal to that in Fig. 2e,f. The *ϕ*=0 orientation of the second-iteration Koch-star particles (Fig. 2e) shows 28 compensated defect pairs. As it is quite easy to observe point defects (or projections of line defects onto the imaging plane), it was not possible to detect any disclination line, running along the upper and lower edge of the first and second-iteration Koch particles. These lines must be depressed into the particle or strongly pinned to the surface, being therefore inaccessible to grabbing by the laser tweezers. The numerical modelling shows that the general 3D morphology of the topological defects for all the Koch iterations 0 to 3 is an integrated combination of the disclination lines with winding numbers +1/2 and −1/2 that join together in a specific order, as shown in Fig. 2a, IV–f, IV and Supplementary Fig. 8, and commented in Supplementary Note 4. Good agreement between experiments and modelling is found. Notably, the exact 3D morphology of the defects—especially at the top and bottom of the particle—is affected by the sharpness of the particle edges and the exact orientation of the particle (Supplementary Fig. 9), as the edges can pin or even locally suppress sections of the defect loops. To generalize, the generation of topological defects and the corresponding topological states are the result of an interplay between geometry and topology, where the fractal surface modulations induce local formation of defect pairs to minimize the elastic distortion of the nematic field.

### Topology of fractal defect states

**Figure 3:**
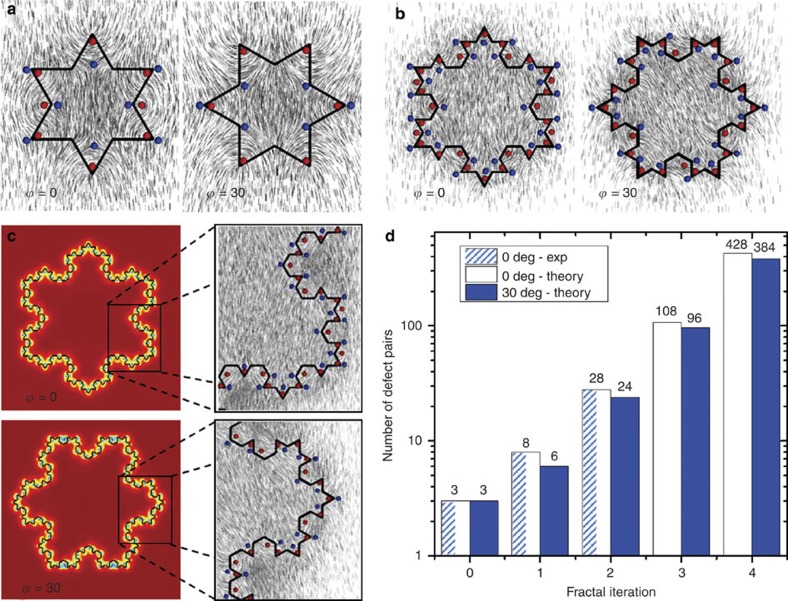
Fractal geometry as generator of defect pairs. (**a**–**c**) Pairs of +1/2 (in red) and −1/2 defects (in blue) and surrounding director field (in grey) as generated by Koch particles of iterations *N*=1–3 at angles *ϕ*=0° and *ϕ*=30° of the particles relative to the far-field undistorted nematic director. The director and defects are plotted in the later mid-plane cross-section of the particle; these indicated 2D defect points are formally just two-dimensional cross-sections of actual 3D defect loops that entangles the whole particle. (**d**) Number of the defect pairs for different iterations as obtained from experiments and numerical modelling. Particles of size *l*_b_/*ξ*=100 are used in the numerical analysis.

Topologically, the considered Koch particles are equivalent to tori, thus having zero total topological charge^24^. Having immersed the particles into a uniformly aligned nematic field (that is, planar nematic cell), therefore, also the net topological charge of all the surrounding defect structure must be equal to zero. Indeed, observing the structure of defects, they are a complex three-dimensional topological structure which effectively consists of multiple mutually fused defect loops that engulf the particle (Supplementary Fig. 8). A quantitative relation between the fractal surface and the generation of topological defects can be established by observing the nematic profile in a selected (x-y) plane that intersects the fractal-modulated surface, as shown in Fig. 3. In this cross-section, which effectively, can be considered as a quasi-two-dimensional nematic, the director is roughly fully in-plane and the topological defects are seen as two-dimensional +1/2 and −1/2 winding number point defects (but are actually cross-sections, which effectively, can be considered as a quasi-two-dimensional nematic, of the three-dimensional defect loop). These +1/2 and −1/2 defects are all observed to emerge in mutually compensating pairs, in all fractal iterations of Koch particles, which assures homogeneous nematic far-field (Fig. 3a–c).

By considering the geometric parameters of the Koch surface, especially the total number of edges which grows as 3 × 4^*N*^ with iteration *N* (*N*=0,1,2,..), the number of the defects pairs for given iteration *N* can actually be determined as a rule, if self-similarity of the nematic response upon changing the iterations is assumed. Accordingly, we find that the number of defect pairs *n* for orientation *ϕ*=0 of Koch particles is equal to 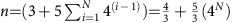 (for *N*>0; and *n*=3 for *N*=0), and for orientation *ϕ*=30° of the particles 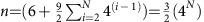 (for *N*>1; *n*=3 for *N*=0 and *n*=6 for *N*=1), growing exponentially with iteration *N*. The number of defect pairs observed in experiments (for iterations *N*=0–2) and in numerical modelling (for iterations *N*=0–4) is shown in Fig. 3d and is in exact agreement with the analytically predicted formula. We should stress that this observed exponential growth of the number of defect pairs actually ends when fractal feature size becomes comparable to the nematic correlation length, as individual defect cores do not form anymore but rather larger regions of reduced degree of order start to emerge.

### The role of particle size and fractal-self-similarity

**Figure 4:**
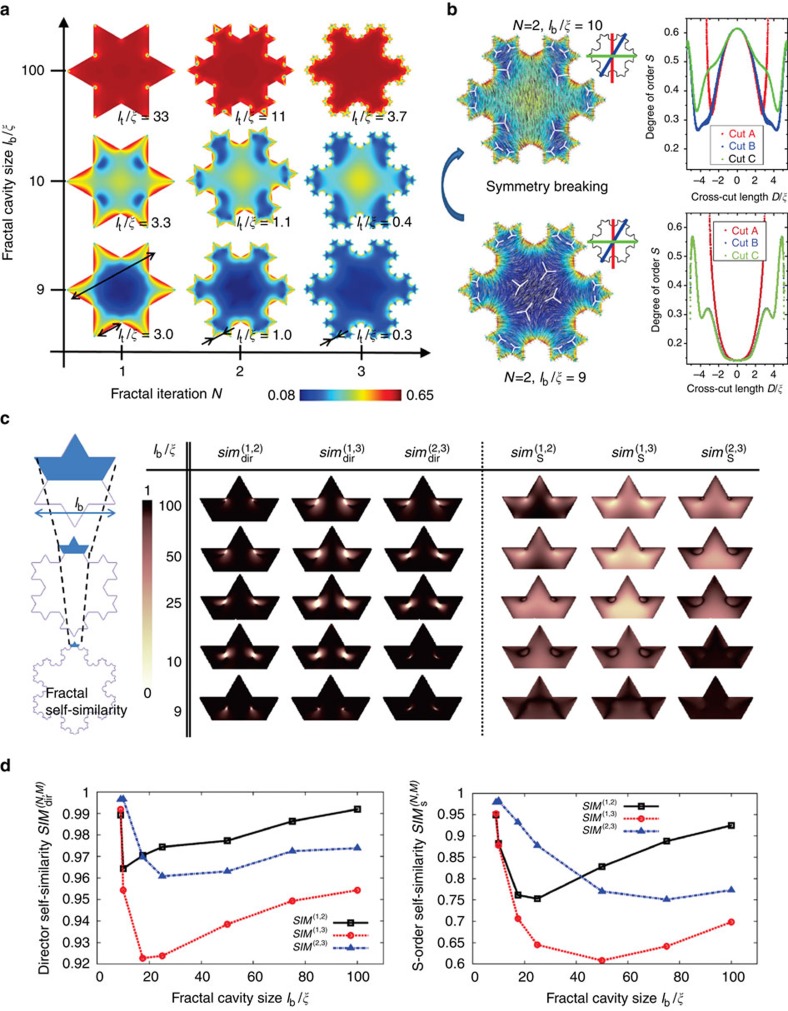
Role of fractal size and fractal self-similarity in Koch cavities. (**a**) Fractal response for different fractal iterations and fractal cavity sizes, shown for nematic degree of order in a cross section of a long Koch particle. (**b**) Change in the symmetry of the nematic profile upon reducing the size of the cavity. Right panels shown the variation of the nematic degree of order across the three cross-cuts indicated in the inset. Note the increase in the symmetry for the *l*_*b*_/*ξ*=9 profile. (**c**) Self-similarity of nematic director and degree of order between iterations 1 and 2, 1 and 3, and 2 and 3, as calculated for different Koch cavity sizes *l*_*b*_/*ξ*. (**d**) Integrated self-similarity of nematic director and degree of order calculated form profiles in **c**, for different Koch cavity sizes.

The fractal topological states depend on the size of the Koch cavities, as shown in Fig. 4a where the particle size (that is, edge length *l*_b_) relative to the nematic correlation length *l*_b_/*ξ* is in our study the main size parameter as the surfaces are taken in the strong (but finite) anchoring regime with surface extrapolation length^22^ *ξ*_S_ generally shorter than *l*_b_ and *ξ* (*ξ*_S_∼1 nm). Nematic correlation length is the elementary scale of nematic when considered at the mesocopic level and describes the ratio between elasticity and bulk order, effectively scaling as 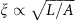, where *L* is the nematic elastic constant and *A* the bulk ordering term^21^. For large particle or fractal feature sizes, the structures are characterized by the formation of pronounced individual defects, with well-determined core regions of reduced nematic degree of order. By increasing the fractal iteration, new defect-antidefect pairs form and the complexity of the fractal nematic pattern increases. The regime of large *l*_b_/*ξ* is actually the regime of the presented experiments and calculations shown in Figs 2 and 3. However, when the particle size (*l*_b_) or the fractal feature size (*l*_t_) become comparable to the nematic correlation length (bottom two rows in Fig. 4a), the molten cores of nematic defects effectively start to overlap, and can fuse into larger regions with reduced nematic degree of order, for example, see large regions of low degree of order in Fig. 4a at *N*=3, *l*_b_/*ξ*=9 (in blue). Effectively, by increasing the fractal iterations, the fractal surfaces start to impose an overly complex frustration on the nematic order, and it becomes locally more energetically favourable for the nematic to reduce degree or order and approach isotropic phase and change the symmetry of ordering within the fractal cavity (see Fig. 4b), which is a process that could be interpreted as fractal nematic melting. Opposite process is known in other systems known as capillary condensation where nematic forms within the isotropic background due to the stabilization from the surface^33^. Experimentally, the observed fractal nematic melting could possibly be realized in systems where geometrical feature sizes can be made of similar size order as the nematic correlation lengths, such as in colloidal nematic liquid crystals^34^.

The fundamental feature of fractals is self-similarity at different length-scales and the Koch geometry (without nematic) is infinitely self-similar upon increasing the fractal iteration. However, for the nematic response—that is, the director and nematic degree of order- we find that they are self-similar only to some approximation and within distinct range of scales. The relative self-similarity of nematic surrounding Koch particles of fractal iterations *N* and *M* (Fig. 4c) is quantified by introducing two self-similarity functions (that is, overlap functions or norms): for the director 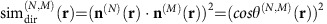, where *θ*^(*N*,*M*)^ is the relative angle between the directors in iterations *N* and *M* at position **r**, and for the nematic degree of order 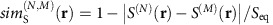 (*S*_eq_ is the bulk nematic degree of order). The two self-similarity functions are constructed to be equal to one if the patterns at different fractal iterations are locally self-similar and become zero if not (for more, please see Supplementary Note 2). The self-similarity functions are calculated for the selected regions, comparing iteration pairs (*N*=1, *M*=2), (*N*=1, *M*=3), and (*N*=2, *M*=3), as shown in Fig. 4c. The patterns of nematic director and nematic degree of order emerge to be well similar over the repeating fractal region, except for close to defect cores where differences at the length scale of the nematic correlation length are observed. Especially, the defects change location relative to the edges. As an even more focused measure of the self-similarity we integrate the self-similarity functions over the considered regions Ω, 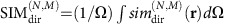 and 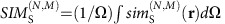, allowing us to simply quantify the relative self-similarity of different iterations, as shown in Fig. 4d. Self-similarity can be further analysed by introducing various forms of correlation functions (Supplementary Note 5; Supplementary Fig. 10).

## Discussion

The general deviation of the nematic response from the full self-similarity emerges to be at the level of several per cent, and decreases with either small or large fractal cavity sizes *l*_b_/*ξ*, which can be explained by two main mechanisms: (i) the presence of the nematic correlation length and (ii) the fundamental uniaxial ordering of the nematic. On one hand, the nematic correlation length affects the exact structure and position of the defect cores; therefore, the nematic director and degree of order are well self-similar only in the regime of either large or small Koch cavities relative to the correlation length, where defect cores are either large or small, subjected to the condition that surface-anchoring regime is not notably different at all these different cavity length scales. Changing the nematic correlation length—that is, either changing the material itself or varying parameters like temperature- will shift the effective window of self-similarity to different physical length scales. On the other hand, the uniaxial ordering breaks the symmetry of the director profiles within the fractal arms (for example see Fig. 4b) making some arms different at different fractal scales and some not. This local loss of self-similarity in distinct fractal regions is the consequence of inherent long-range nematic elasticity which causes that the difference in the profiles originating from the roughly uniform nematic region (in our case in the center of the cavity) proliferates into multiple fractal regions and iterations. Possibly, such inherent imprinting of uniaxial order and loss of symmetry could be enhanced or suppressed by using fractal patterns of different geometry. Also, interesting to explore would be the role of the surface anchoring and its effects on the self-similarity. Any variation of the surface-anchoring strength would introduce another length scale into the system—the surface extrapolation length—leading to further complex interplay between surfaces, bulk nematic elasticity and the fractal geometry.

In summary, we have demonstrated fractal nematic colloids as novel materials, which are a distinct realization of the coupling between the fractal order and uniaxial nematic vector-type ordering. Koch colloidal particles are produced via nano-printing technique in the form of hollow prisms with fractal belt surface and used as inner and outer confinement for the nematic field. The formation of fractal topological states characterized by locally compensating pairs of topological defects is shown, as governed by the local geometry of the fractal surface and its iteration, and less by the topology. This is analogous to the recent observation of topological states on a fibre with genus *g*=0, which can carry any odd number of topological defects with a total charge of −1 (ref. 35). The number of fractal generated defect pairs are shown to follow exponential-law series, reaching already ∼100 defect pairs at fractal iteration 3. The ratio between the fractal feature size and the nematic correlation lengths is shown to crucially affect the exact response of the nematic, also conditioning the size-window and number of succeeding fractal iterations that actually can be realized with given materials. Basic self-similarity functions between different fractal iterations are introduced for the nematic director and the degree of order, and used to quantify the fractal self-similarity of the nematic pattern. More broadly, this work demonstrates the response of effectively elastic vector-type fields to fractal stimulus or surfaces, resulting in a broad series of topological states – that is, field conformations governed by complex fractal self-similar patterns of topological defects- which are stabilized by the fractal geometry. Finally, this work is a contribution towards novel stimulus responsive soft materials and can prove relevance in diverse fields ranging from confined active nematic systems to multi-scale photonics and lasing.

## Methods

### Experiments

Koch particles were produced by direct laser writing using a commercially available Photonics Professional (Nanoscribe Gmbh) and a photoresistive gel-like photoresist IPG (Nanoscribe). The particles were later treated with an aqueous solution of DMOAP (ABCR GmbH), which enforces strong homeotropic anchoring on the surfaces of the Koch nematic colloids. The colloidal particles were then immersed in a low birefringent mixture of CCN 47 (50%) and CCN 55 (50%) (Nematel GmbH), which is nematic at room temperature. The suspension was then placed in a glass cell of thickness 30 μm with strong planar anchoring at the boundaries. The system was then observed using polarized optical microscopy, where the angle of the analyser with respect to the polarizer was changed, thus improving the contrast of the disclination lines in the images.

### Numerical modelling

The numerical modelling of the system was performed using the Landau-de Gennes free-energy expression which is written in powers of a local symmetric traceless tensor order parameter, *Q*_*ij*_, and its derivatives, ∂_*k*_*Q*_*ij*_. The tensor order parameter was used to characterize nematic orientational order about the local average molecular directions, called director, **n**, and the local degree of molecular order along the directors, called nematic degree of order *S*. The total free-energy functional is written as follows


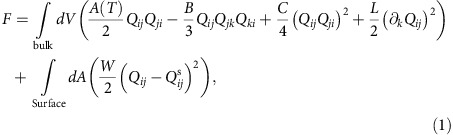


in which *A*, *B* and *C* are material dependent parameters and *A* is also taken to be linearly dependent on temperature. *L* is the elastic constant and *W* is the anchoring constant. To impose homeotropic anchoring condition on the colloidal surfaces we take 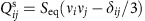 in which **ν** denotes normal vectors on the colloidal surfaces. In the absence of external constraints there is a uniform bulk nematic with the equilibrium scalar order parameter equal to 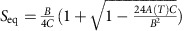 and the correlation length of the liquid crystal in this mean field theory is given as 
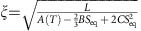
. The following material parameters are used: *A*=−0.07 × 10^5^ J m^−3^, *B*=3.6 × 10^5^ J m^−3^ *C*=3.0 × 10^5^ J m^−3^, *L*=1.0 × 10^−11^ N *W*=1.0 × 10^−2^ J m^−2^. On the basis of these parameters we have ξ=10 nm and *S*_eq_=0.653. The length scale of the fractal colloids and cavities is rescaled as *l*_b_/ξ and takes values in the range from 9 to 100. The free energy is numerically minimized by a finite-element method^36^,^37^.

### Data availability

The data that support the findings of this study are available from the corresponding author upon request.

## Additional information

**How to cite this article**: Hashemi, S. M. *et al*. Fractal Nematic Colloids. *Nat. Commun.* **8**, 14026 doi: 10.1038/ncomms14026 (2017).

**Publisher's note**: Springer Nature remains neutral with regard to jurisdictional claims in published maps and institutional affiliations.

## Supplementary Material

Supplementary Information Supplementary Figures 1-10, Supplementary Notes 1-5 and Supplementary References
