# Supplementary material for: Fractal nematic colloids
Source: Nat Commun. 2017 Jan 24;8:14026. doi: 10.1038/ncomms14026 (PMC5286210; doi:10.1038/ncomms14026)
Supplement: Supplementary Information — Supplementary Figures 1-10, Supplementary Notes 1-5 and Supplementary References [file ncomms14026-s1.pdf]

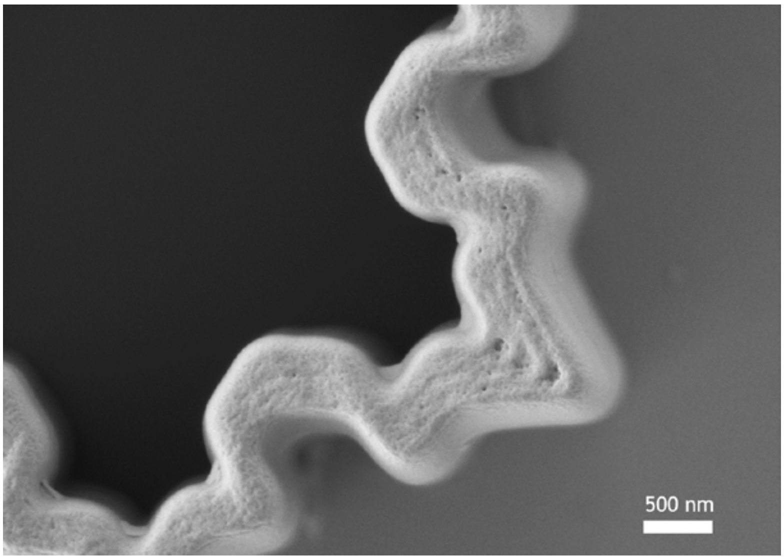

Supplementary Figure 1 | SEM pictures of Koch fractal colloidal particles. The particles were drawn line by line in a gel like photoresist therefore we can still see individual lines on the corners of the particle.

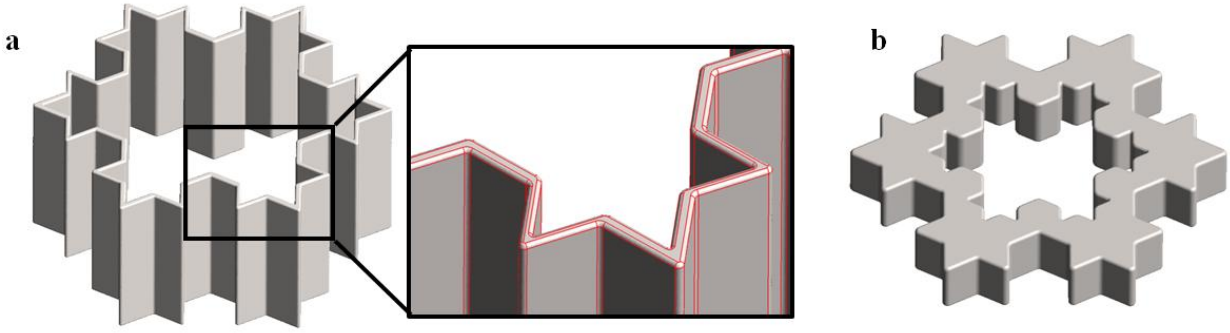

Supplementary Figure 2 | Geometric construction of Koch particles. a, Zoomed-in presentation of the rounded edges of the Koch iteration 2 as a typical example of all Koch particles which are numerically modeled. **b,** Koch particle of iteration 2 with different geometric parameter values than those of the modeling showing the tunable geometric parameters of the modeled particles.

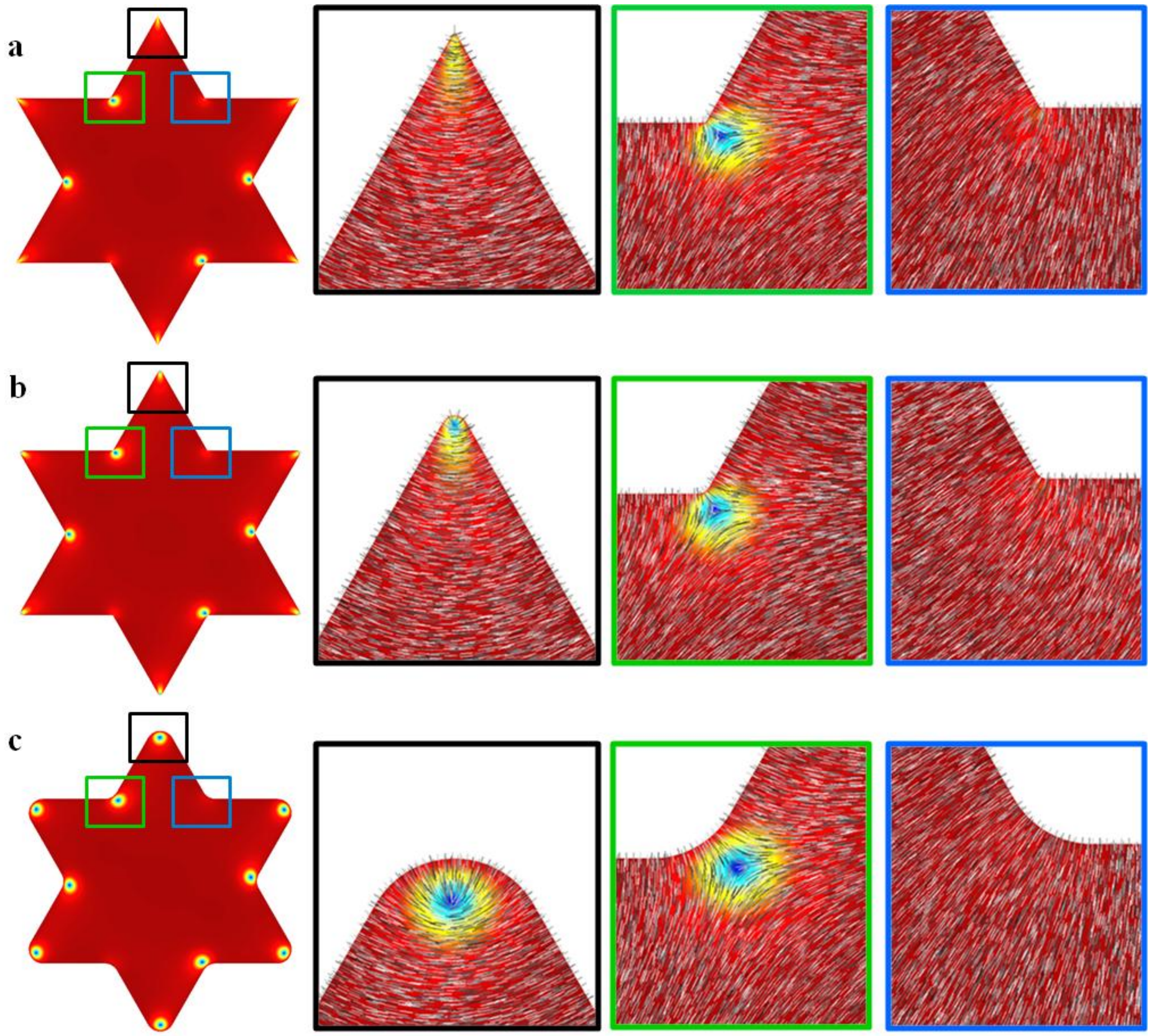

Supplementary Figure 3 | The effect of edge roundedness on the nematic profile. A Koch cavity of iteration 1 with $l_{\mathrm{b}}/\xi = 100$ and three different curvature radii **a,** 0 (sharp edges), **b,** $l_{\mathrm{b}}/140$, and **c,** $l_{\mathrm{b}}/20$ is considered. The first panel shows the contour plot of the scalar order parameter across the cross section of the cavities. The three last panels illustrate three zoomed-in selected parts of the cavities respectively containing a +1/2 defect, a -1/2 defect and a defect-free region. The same color bar as in Fig. 4a is used for the scalar order parameter presentation.

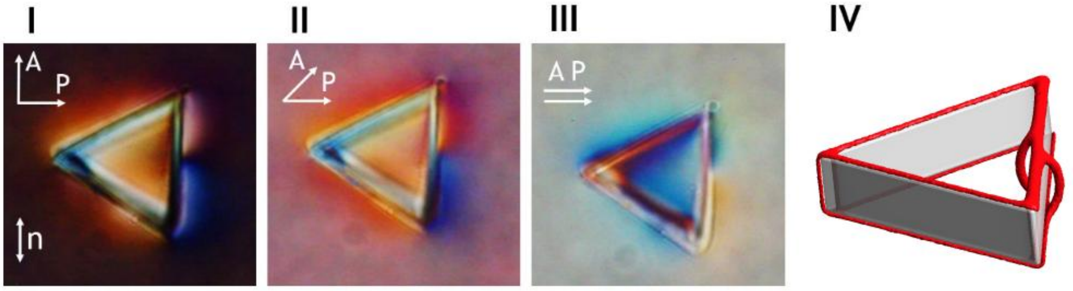

Supplementary Figure 4 | Characterization of the disclination line of the 0th iteration Koch nematic colloid. (I-III), Polarized images of 0th iteration Koch nematic colloid. By rotating the analyzer one can clearly resolve a closed disclination line winding along the upper edge of the particle because of the change in contrast in the image. On the other hand one can clearly see that the disclination line doesn't follow the upper edge of the defect on the left corner of the particle. **(IV),** Numerical representation of disclination lines around 0th iteration Koch nematic colloid. The 3D view of the defect structure in which the topological defect cores are visualized by an isosurface of $S = 0.46$.

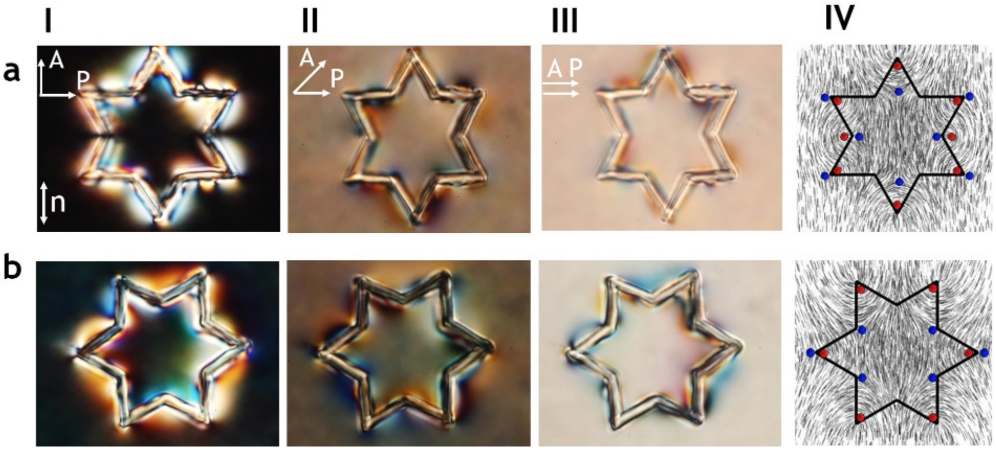

Supplementary Figure 5 | Localization of defect lines along 1st iteration Koch nematic colloid. Polarized images of 1st iteration Koch nematic colloid at two orientations relative to the rubbing direction **a** and **b**. Panels I-III are experiments and Panel IV is results of numerical modelling.

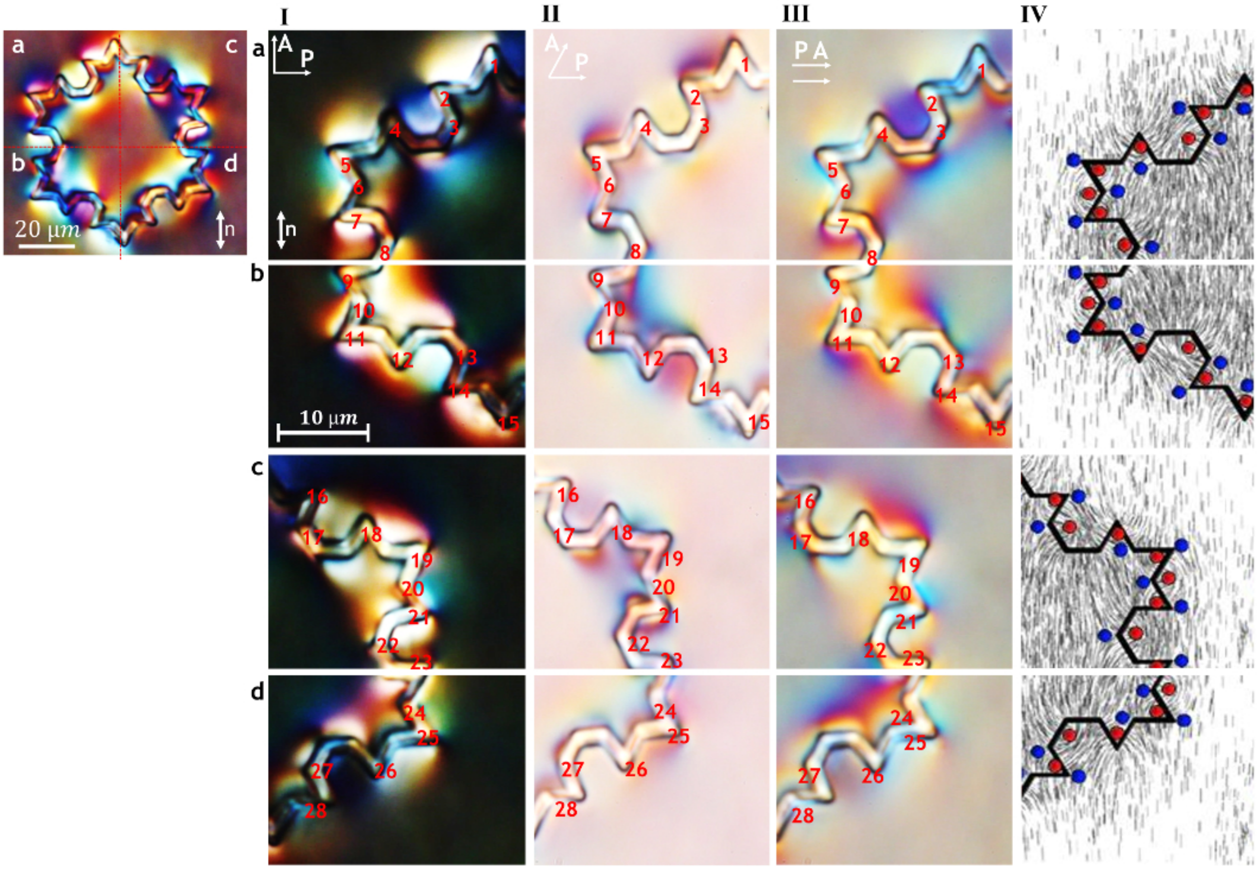

Supplementary Figure 6 | Study of the defect location in the 2nd iteration Koch nematic colloid at angle 0°. a-d, individual quadrants of the Koch nematic colloid observed **I-III,** with different orientation of the analyzer and Panel **IV** is results of numerical modelling**.**

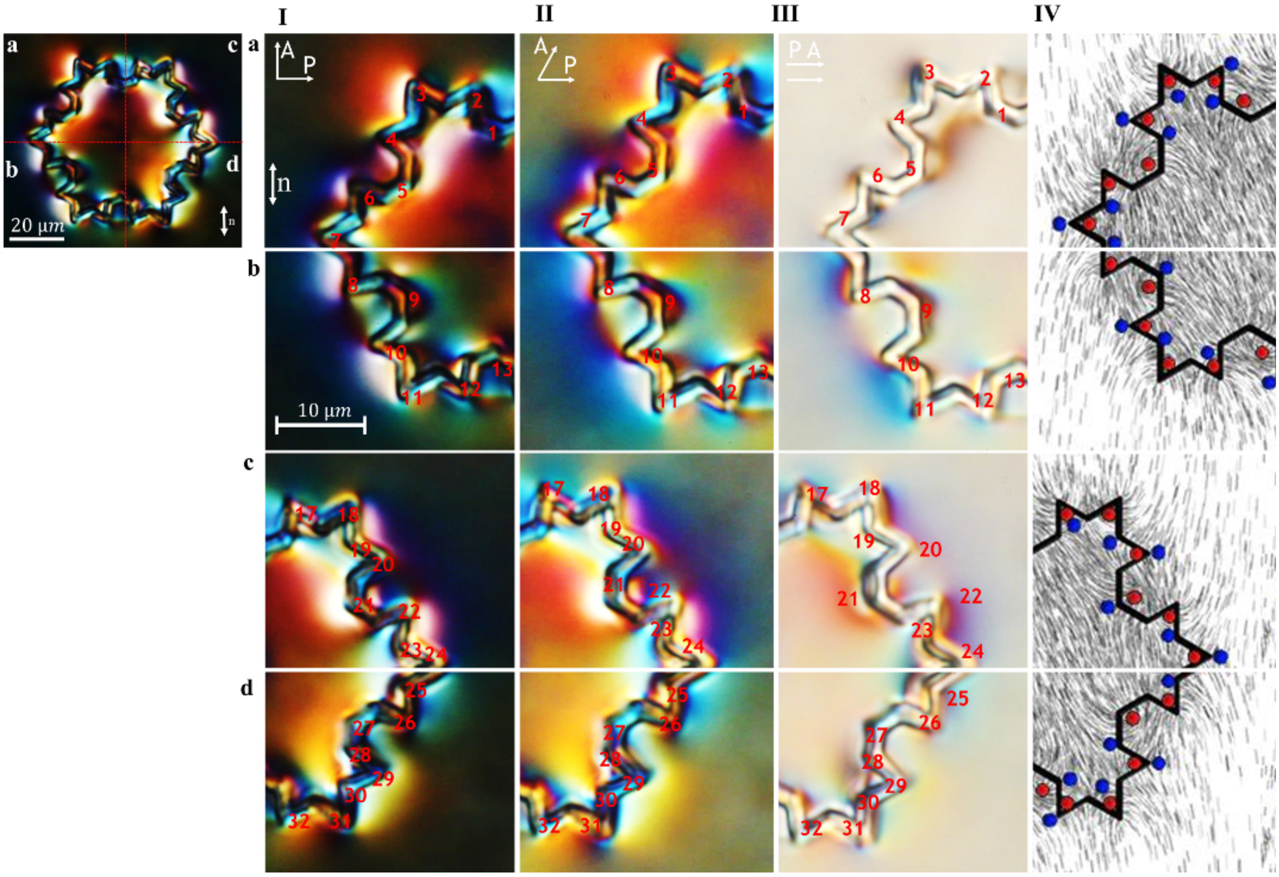

Supplementary Figure 7 | Study of the defect location in the 2nd iteration Koch nematic colloid at angle 30°.

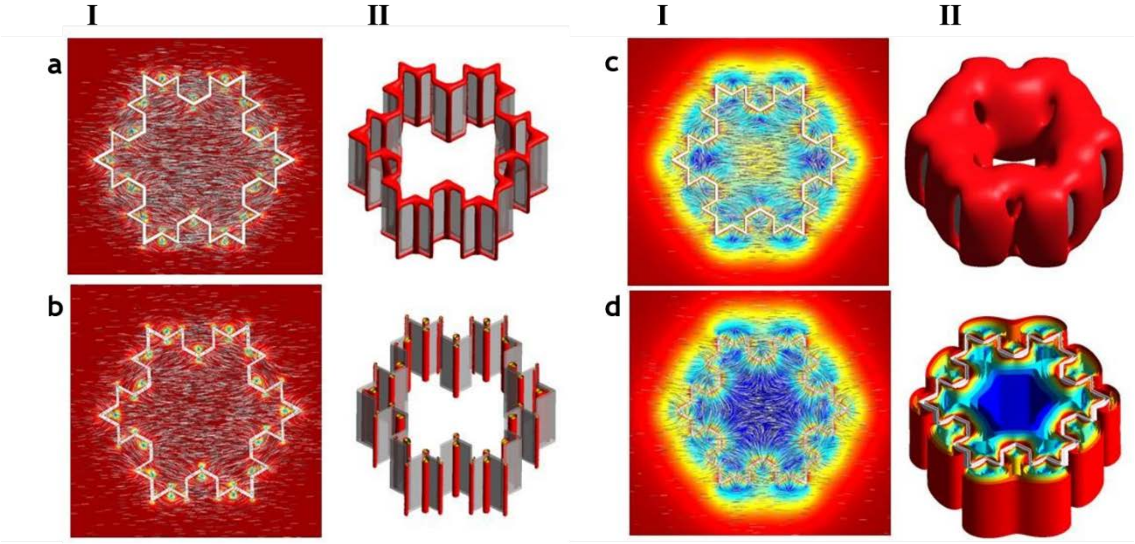

Supplementary Figure 8 | Three-dimensional profile of nematic defects for Koch colloidal particles and (infinitely) long Koch cavity. Each section contains two parts; **I,** a 2D contour plot of the scalar order parameter and director field across the midplane of the particles in the $x-y$ coordinate plane. The same color bar as Fig. 4a is used for the scalar order parameter, **II,** The 3D view of the defect structure in which the topological defect cores are visualized by an isosurface of $S = 0.46$. **a,** Koch particle with $l_b/\xi = 100$ and $h = l_b/2$. **b,** Koch cavity with $l_b/\xi = 100$. **c,** Koch particle with $l_b/\xi = 9$ and $h = l_b/2$. **d,** Koch cavity with $l_b/\xi = 9$.

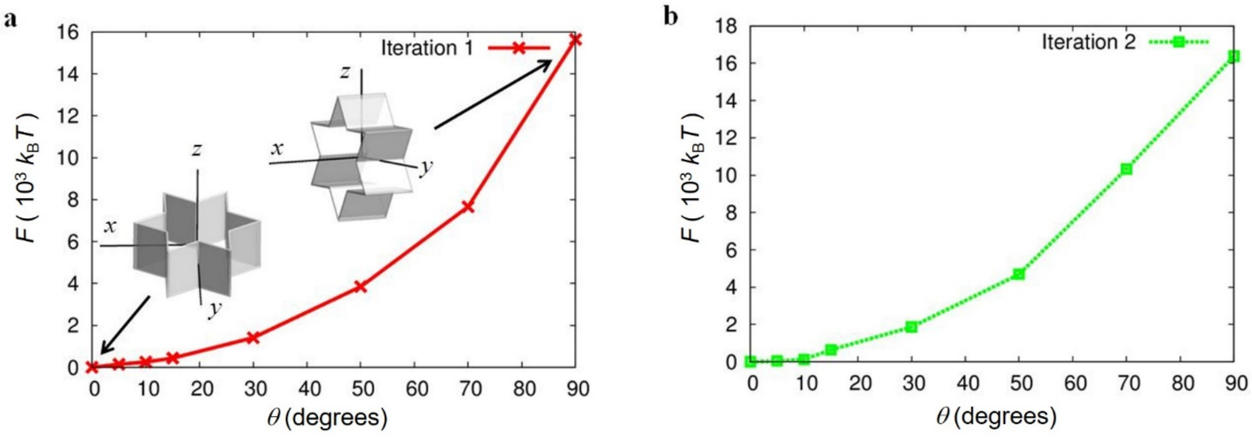

Supplementary Figure 9 | Equilibrium orientational state of the Koch particle symmetry axis. Free energy F with respect to the polar angle θ, which is the angle between the particle's rotational symmetry axis and the z axis are shown. Energy graphs are shown for Koch particles of size $l_\mathrm{b}/\xi = 100$ for **a,** iteration 1 and **b,** iteration 2.

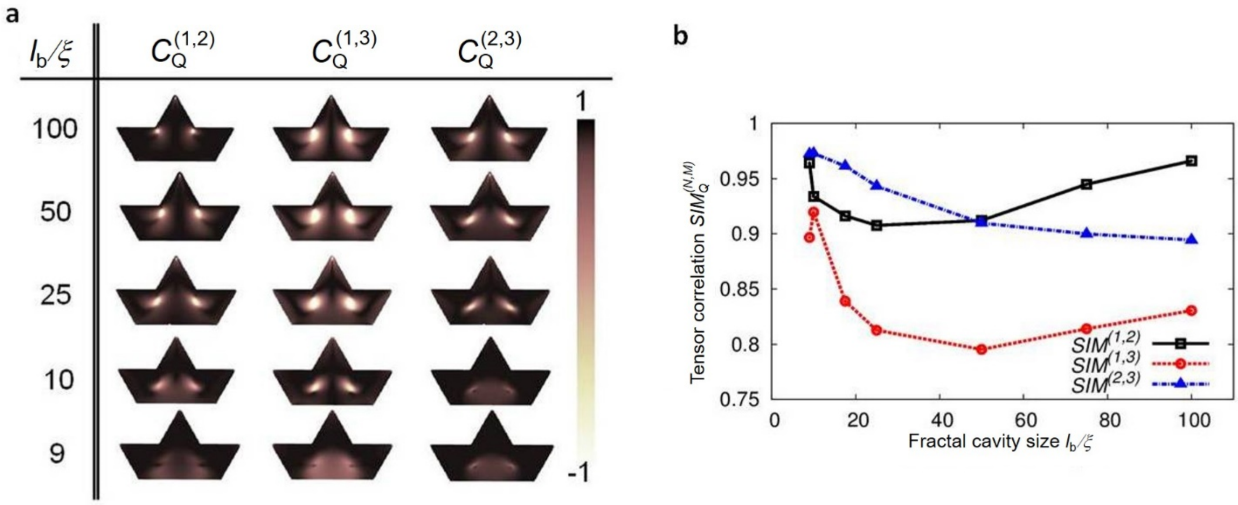

Supplementary Figure 10 | Correlation function as a measure of self-similarity. a, Local value of $C_\mathrm{Q}^{(N,M)}(\mathbf{r})$ between nematic tensor order parameter of iterations 1 and 2, 1 and 3, and 2 and 3, as calculated for different Koch sizes l_b/ξ . **b,** Integral values for self-similarity of nematic tensor order parameter with respect to Koch cavity sizes.

Supplementary Note 1
Experimental preparation of the Koch colloidal particles, surface treatment of particles and cell preparation

Koch colloidal particles were produced using a commercially available machine Photonics Professional (Nanoscribe GmbH), and the photoresist IPG (Nanoscirbe). The system focuses high intensity 780 nm laser light in the photo resistive material, where due to the two photon absorption process the material is polymerized. With our parameters the typical polymerization volume around the focus, called the voxel, is less than 150 nm wide and 400 nm high. The particles were manufactured line by line in 4 layers of 150 nm which corresponds to 600 nm wall thickness. After the polymerization process we use a commercially available SU-8 developer (PGMEA) to wash off the unpolimerized section of the photoresist. Some particles were powder coated and observed with a Scanning Electron Microscope shown in Supplementary Fig. 1.

We imposed homeotropic anchoring conditions on the surfaces of the Koch colloidal particles. Therefore we plasma cleaned the particles, which activates the particle surfaces. The particles were than submerged in an aqueous solution of DMOAP (c=0.5%) and after rinsing baked for one hour at 110°C, which hardens and crosslinks the DMOAP to the surface. We used a low birefringent, mixture of two liquid crystals CCN 47 and CCN 55, which exhibits the nematic phase at room temperature. The mixture of the liquid crystal was placed on top of the particles which were then detached into the liquid crystal with help of a fine needle. The liquid crystal mixture and Koch colloidal particles were then transferred into a glass cell with planar anchoring conditions imposed on the substrates. The final cell thickness was 30 μm compared to the Koch particle size of 8 μm.

Supplementary Note 2
Numerical modelling of Koch nematic colloids

To model the Koch nematic colloids we use numerical minimization of the Landau-de Gennes (LdG) free energy with Finite Elements Method. The free energy consists of three parts: a temperature-dependent bulk free energy, a bulk free energy for the elasticity of the nematic medium, and a surface free energy to impose a preferred anchoring of the directors on the colloidal surfaces

$$F = \int_{bulk} dV\left(\frac{A(T)}{2}Q_{ij}Q_{ji} - \frac{B}{3}Q_{ij}Q_{jk}Q_{ki} + \frac{C}{4}(Q_{ij}Q_{ji})^2 + \frac{L}{2}(\partial_k Q_{ij})^2\right) + \int_{Surface} dA\left(\frac{W}{2}(Q_{ij} - Q_{ij}^{s})^2\right)$$

By adopting the same material parameters as in Ref. 1 for A, B, and C, we fix the temperature of the model system in the state of stability of nematic phase. L is an elastic constant in one elastic constant approximation for modeling the elasticity of the nematic medium. The surface energy functional is taken over all the colloidal surfaces to impose a strong homeotropic surface anchoring with the anchoring strength W. In case of a finite-sized Koch particle immersed in a uniform nematic field, we model the uniform field by using a cubic box with a fixed anchoring condition in the x direction. In case of modeling the long Koch cavity (in Fig. 4), we consider a finite-sized cavity with homeotropic anchoring at the lateral face and free bulk condition at the top and bottom faces. We checked that the free bulk condition at the top and bottom faces gives the same result as a periodic condition. These conditions give the same results because of a translational symmetry along the symmetry axis of the cavity. We chose the free bulk condition in order to reduce the numerical minimization time.

Custom-developed finite element method is developed for the numerical minimization of the free energy[2-4]. We use the three-dimensional (3D) finite element mesh generator Gmsh[5] that decomposes the nematic bulk of the system into tetrahedral mesh elements and the surface of the colloidal particle into triangular elements. We always set the mesh size to be much smaller than the correlation length of the model system. In this way the mesh generator allocates fine mesh sizes that are smaller than the correlation length near the colloidal surfaces where the topological defects are located. By allocating arbitrary initial director fields to the mesh grid, the numerical minimization of the free energy is done using the Numerical Recipes library[6].

To calculate the self-similarity functions director $sim_{\mathrm{dir}}^{(N,M)}(\mathbf{r})$, $sim_{\mathrm{S}}^{(N,M)}(\mathbf{r})$, $SIM_{\mathrm{dir}}^{(N,M)}$ and $SIM_{\mathrm{S}}^{(N,M)}$, we need a one-to-one correspondence (bijection) between the mesh points of the two configurations. The Koch cavities have a translational symmetry along their symmetry axes. Therefore, to do self-similarity checks, information on a 2D cross-section of the Koch cavities is sufficient. The Gmsh allocate different mesh point distributions to each configuration. In order to construct a one-to-one correspondence between the mesh points of the two configurations and subsequently make the self-similarity comparison possible, we map the Gmsh mesh of the two configurations into the same square mesh grids. This is a kind of coarse graining in which to each point of the new square mesh grid the average order parameter of the Gmsh mesh grid points in its near neighbors is allocated.

The particle shapes are allocated as shown in Supplementary Fig. 2a, where notably the particle edges are rounded. The particle's shape is declared via tunable variables in such a way that the geometric parameters, like the side length, height, thickness and curvature radii of the particles, can be determined independently from each other. Supplementary Fig. 2b is presented as a possible example.

Koch particles were modelled with rounded edges, to approach experimental simulation and avoid strong non-realistic pinning of defects to sharp edges. In Supplementary Fig. 3 the numerical modeling results for three different edge roundedness of a Koch cavity of iteration 1 with size $l_{\mathrm{b}}/\xi = 100$ are shown. The rounded edges in the inner part of the cavities have either an acute angle of 60°or an obtuse angle of 240°. In the interior of the cavities, very close to all the edges with an acute angle of 60° one defect with winding number +1/2 is formed. The defects with the winding number -1/2 are formed farther from the acute edges more closely to the edges of 240°. Supplementary Fig. 3 shows that by making the edges more rounded the defect structures do not change and just the defect cores get more pronounced/wider and move a little farther from the surface into the nematic bulk.

Supplementary Note 3
Characterization of the defect structure

We observed the behavior of the nematic director field around the Koch colloidal particles by using conventional polarizing microscopy. The experimental set up is as follows. We use a bright halogen lamp to achieve high illumination of our samples. A polarizer was placed before our sample to illuminate our sample with linearly polarized light, where the angle of polarization is set to be perpendicular to the rubbing direction of our sample. After the light passes and interacts with the sample we used a freely rotating analyzer which allowed us to increases the contrast of the disclination lines in the image.

The zero-iteration Koch star particles always orient themselves with one side perpendicular to the rubbing direction. By rotating the analyzer at fixed polarizers shown in Supplementary Fig. 6 one can clearly resolve that defects in the corners are actually pairs of defects with opposite topological winding and charge. Each of the three pairs of defects in each corner of the triangle therefore compensates the winding, giving total winding zero, as expected for the total charge of a torus. The

walls of the real particles have a final thickness which causes the disclination line sometimes to slide to the side which is shown in Supplementary Fig. 4-IV.

The first iteration Koch particles has two stable configurations. In the first, one corner is pointing in the direction of the director field, shown in Supplementary Fig. 5-a. The second stable configuration is characterized by the fact that one corner is pointing perpendicular to the direction of the director field shown in Supplementary Fig. 7-b. By rotating the analyzer the contrast of the disclination lines increases so one can clearly resolve the areas with high optical distortion as topological defects which are pinned to the edges and corners of the particles, shown in Supplementary Fig. 5(a,b)(I-III). Numerical simulation with topological defects of different winding marked with different colors are shown in Supplementary Fig. 5(a,b)(IV).

The second iteration of Koch particles also exhibits two stable configurations. The first iteration Koch particles has two stable configurations. In the first, one corner is pointing in the direction of the director field, shown in Supplementary Fig. 6. The second stable configuration is characterized by the fact that one corner is pointing perpendicular to the direction of the director field shown in Supplementary Fig. 7. By slicing the image in 4 quadrants and then enlarging them allows us to see the defect structure from up close. By rotating the analyzer the contrast of the disclination lines increases so one can clearly resolve the areas with high optical distortion as topological defects which are pinned to the edges and corners of the particles, shown in Supplementary Fig. 6-7(a-d)(I-III). Numerical simulation with topological defects of different winding marked with different colors are shown in Supplementary Fig. 6-7(a-d)(IV).

Supplementary Note 4

Three-dimensional structure of nematic defects surrounding Koch particles and equilibrium orientation of Koch particles

Supplementary Fig. 8a and 8c show the three-dimensional profiles of the nematic director surrounding Koch colloidal particles, notably including three-dimensional structure of defects, as in relation with Fig. 2. Indeed, modelling results in Fig 2 (iteration 2) are taken as direct cross-section from Supplementary Fig. 8a. The topological structure, spatial distribution and core size of the defects depend not only on the geometric shape of the colloidal surface but also on the relative size of the particle to the two material characteristic lengths; extrapolation and correlation lengths. For the micron-sized Koch particles the topological defect cores form in localized regions close to the surface of the particles (Supplementary Fig. 8a). By reducing the size of the particles to $l_{\mathrm{b}}/\xi = 9$, the upper and lower edges pin all the defects near the surfaces and the vertical defect lines in Fig. 2e (Supplementary Fig. 8c) widen in a little farther position than those of a particle with $l_{\mathrm{b}}/\xi = 100$.

Supplementary Fig. 8b and 8d show the nematic profile surrounding Koch colloidal particles modelled as long cavities, i.e. (infinitely) long tubes, which directly relates to analysis in Fig. 4. In such modelling, in 3D, the defects are seen as long +1/2 and -1/2 disclinations following the fractal surface, with no effect of top or bottom on the exact symmetry in the center of the cavity (which can emerge when modelling particles –not cavities- which are thin relative to the edge length l_{b}).

The Koch particles stand with their rotational symmetry axis perpendicular to the uniform field. This equilibrium orientation is both numerically checked (Supplementary Fig. 9) and experimentally observed. In Supplementary Fig. 9 the numerical modeling results for the free energy with respect to θ for the iterations 1 and 2 and the particle size $l_{\mathrm{b}}/\xi = 100$ are shown. These results show one global minimum in the free energy graphs in $\theta = 0$ for both particle iterations.

Supplementary Note 5
Correlation function expression to quantify self-similarity

The self-similarity quantifying functions are not unique and self-similarity can be analysed also by using correlation functions, especially since the correlation function theory is a well-known concept in a variety of research fields. The Q tensor correlation function between the fractal iterations N and M is introduced as:

$$C_{\mathrm{Q}}^{(N,M)}(\mathbf{r}) = h_{\mathrm{Q}}^{(N,M)}(\mathbf{r}) \Big/ \sqrt{|h_{\mathrm{Q}}^{(N,N)}(\mathbf{r})| \times |h_{\mathrm{Q}}^{(M,M)}(\mathbf{r})|}$$

where

$$h_{\mathrm{Q}}^{(N,M)}(\mathbf{r}) = Tr\left(\mathbf{Q}^{(N)}(\mathbf{r}) - \frac{1}{\Omega}\int \mathbf{Q}^{(N)}(\mathbf{r})d\Omega\right)\left(\mathbf{Q}^{(M)}(\mathbf{r}) - \frac{1}{\Omega}\int \mathbf{Q}^{(M)}(\mathbf{r})d\Omega\right)$$

and the integrals are over the region Ω shown in Supplementary Fig. 10. $C_{\mathrm{Q}}^{(N,M)}(\mathbf{r})$ has values in the range of $[-1,1]$. The extreme numbers 1 and -1 respectively indicate the correlated and anticorrelated and 0 noncorrelated variables. Supplementary Fig. 10a shows the correlation function for the same selected regions as shown in in Fig.4c, comparing iteration pairs (N=1, M=2), (N=1, M=3), and (N=2, M=3). To obtain a measure of self-similarity we integrate the correlation function, $SIM_{\mathrm{Q}}^{(N,M)} = \frac{1}{\Omega}\int C_{\mathrm{Q}}^{(N,M)}(\mathbf{r})d\Omega$, over the considered region Ω (Supplementary Fig. 10b).

Supplementary References

1. Olmsted, P. D. & Goldbart, P. M. Isotropic-nematic transition in shear flow: State selection, coexistence, phase transitions, and critical behavior. *Phys. Rev. A* **46**, 4966-4993 (1992).
2. Fukuda, J., Stark, H., Yoneya, M. & Yokoyama, H. Interaction between two spherical particles in a nematic liquid crystal. *Phys. Rev. E* **69**, 041706 (2004).
3. Mozaffari, M. R., Babadi, M., Fukuda, J. & Ejtehadi, M. R. Interaction of spherical colloidal particles in nematic media with degenerate planar anchoring. *Soft Matter* **7**, 1107-1113 (2011).
4. Hashemi, S. M. & Ejtehadi, M. R. Equilibrium state of a cylindrical particle with flat ends in nematic liquid crystals. *Phys. Rev. E* **91**, 01250310 (2015).
5. Geuzaine, C. & Remacle, J. F. Gmsh: A 3-D finite element mesh generator with built-in pre- and post-processing facilities. *Int. J. Numer. Meth. Eng.* **79**, 1309-1331 (2009).
6. Press, W. H., Teukolsky, S. A., Vetterling, W. T. & Flannery, B. P. *Numerical Recipes* (Cambridge University Press, 1992).
